# Effects of Physically Active Learning (PAL) Interventions on Physical Fitness, Physical Activity, and Sedentary Behavior in Primary School Children: A Systematic Review

**DOI:** 10.3390/children13091267

**Published:** 2026-09-18

**Authors:** Josip Burušić, Mario Baić, Nebojša Trajković, Damir Pekas, Tihomir Vidranski

**Affiliations:** 1 Institute of Social Sciences Ivo Pilar, 10000 Zagreb, Croatia; josip.burusic@pilar.hr; 2 Department of General and Applied Kinesiology, Faculty of Kinesiology, University of Zagreb, 10000 Zagreb, Croatia; mario.baic@kif.hr (M.B.); damir.pekas@kif.hr (D.P.); 3 Faculty of Sport and Physical Education, University of Niš, 18000 Niš, Serbia; trajcevolley83@gmail.com

**Keywords:** physical activity, physical activity learning, sedentary behavior, children, primary school

## Abstract

**Highlights:**

**What are the main findings?**

- PAL interventions most consistently increased physical activity during targeted academic lessons, whereas effects on whole-school-day, daily, and weekly physical activity remain unclear because of substantial heterogeneity in intervention protocols, measurement instruments, outcome definitions, and assessment periods.
- PAL generally reduced or replaced sedentary classroom behaviour with walking, standing, or sit-to-stand transitions; effects on physical fitness were mixed, with improvements in selected strength, motor-competence, and endurance outcomes but no consistent effects on BMI.

**What is the implication of the main finding?**

- PAL should be implemented as a complementary component of a broader school-based physical-activity strategy that includes physical education, active recess, active commuting, and extracurricular activity.
- Future research should use standardized PAL dose and outcome-reporting frameworks, longer follow-up, and direct measures of clinical health and health-related quality of life.

**Abstract:**

**Background:** Insufficient physical activity has become a significant public health problem. Currently, physical education classes alone are insufficient to meet the physical activity levels recommended by the World Health Organization. Physically Active Learning (PAL), as a novel curriculum, promotes physical activity by teaching new knowledge in various school subjects. **Objective:** To systematically evaluate the effects of PAL interventions on primary school students’ physical fitness, physical activity, and sedentary behavior, characterize the key features of PAL interventions, identify research gaps, and provide evidence-based recommendations for practice. **Methods:** Following the PRISMA guidelines, four electronic databases—Web of Science, ProQuest, Ebsco, and Embase—were searched, and English-language controlled intervention studies that met the PICOS eligibility criteria were included. Two researchers independently screened the literature and extracted and cross-checked the data. Results: This study included 17 studies from 8 countries published between 2009 and 2025. These studies involved primary school students aged 6 to 12. PAL most consistently increased MVPA (moderate-to-vigorous physical activity) or number of steps during the targeted academic lesson, whereas effects on whole-school-day, daily, or weekly physical activity were inconsistent. Lesson-level sedentary behaviour generally decreased or was replaced by walking, standing, or sit-to-stand transitions, but effects on whole-day sedentary time were mixed. Physical fitness interventions yielded varied outcomes, demonstrating significant improvements in upper body strength and explosive muscle strength and endurance, while showing inconsistent effects on aerobic capacity, and no significant changes in BMI or agility indicators. **Conclusions:** Our findings indicate that PAL has the potential to improve students’ physical activity levels in the classroom. However, its effects on physical fitness indicators remained equivocal across evaluated outcomes. PAL should be considered part of a broader school strategy to promote physical activity rather than as a standalone approach. To maximize health benefits, stronger integration of PAL with active recess, active commuting, and structured physical education is recommended.

## 1. Introduction

Insufficient physical activity has become a major public health issue, with nearly one-third of adults worldwide failing to meet recommended levels of physical activity [1]. The majority of adolescents also fail to meet the recommended levels of physical activity [2]. Improving physical fitness, increasing physical activity levels, and reducing sedentary behavior have long been considered primary objectives of school physical education curricula worldwide. Evidence suggests that lack of exercise is a significant cause of most chronic diseases, and that exercise can prevent or delay chronic diseases [3]. As the most widely accessible public health platform, schools have great potential to promote students’ physical health, where improvement of their curriculum and teaching methods is needed.

The school setting offers considerable opportunities to support children’s health and promote regular physical activity throughout the school day. However, due to the structure of the school curriculum, students spend most of school time sitting and listening to lectures, which further contributes to insufficient physical activity [3]. This represents a marked shift from preschool education, where children’s daily activities are typically more strongly oriented toward physical activity and learning through movement. Although physical education classes are essential, they alone may be insufficient to enable students to meet the WHO-recommended levels of physical activity or substantially reduce sedentary time during the school day [3]. Evidence suggests that physical activity may be positively associated with students’ academic performance, while reducing opportunities for physical education may not be beneficial for children’s overall health [4]. In this context, classroom activities and routines may influence the opportunities students have to move and the amount of sedentary behaviour they accumulate during school hours. Consequently, greater attention may be warranted to the ways in which classroom time can be used to provide additional opportunities for physical activity within the school day.

In recent years, Physically Active Learning (PAL) has been promoted globally to increase the total amount of physical activity of students during school time. It is often used as part of the whole-school teaching approach [5]. This is a new type of curriculum that promotes physical activity by teaching new knowledge in various school subjects through the use of physical activity or exercises, with secondary aims to increase daily moderate-to-vigorous physical activity (MVPA) and reduce sedentary time [6]. PAL interventions take many forms and can be directly related to the current learning task or unrelated to the task. They can be carried out indoors or outdoors, resulting in a lack of a unified operational definition and standardized implementation method [7].

Previous systematic reviews have investigated the associations between PAL and cognitive function, academic performance, and physical activity levels [6,7,8,9,10]. One study showed that classroom physical activity has a certain effect on improving academic performance, but no significant effect was found on cognitive function [8]. In addition, classroom teaching that incorporates physical activity can improve students’ physical activity levels and may have a positive impact on learning and health [9,10]. While these studies have provided important insights, comprehensive reviews specifically targeting the effects of PAL interventions on physical fitness, physical activity, and sedentary behavior in primary school students remain limited.

Therefore, this systematic review aims to comprehensively evaluate the impact of PAL interventions on physical fitness, physical activity, and sedentary behavior in primary school students. Furthermore, this review explains the key characteristics of existing PAL interventions, identifies research gaps, and provides evidence-based recommendations for practice and future research.

## 2. Materials and Methods

This research was conducted as part of the institutional research project EDUFIT-HR at the University of Zagreb Faculty of Kinesiology, funded by the European Union—NextGenerationEU.

### 2.1. Sample of Analyzed Studies

#### 2.1.1. Eligibility Criteria (PICOS)

The review question was structured according to the PICOS framework (Table 1). The review included controlled intervention studies involving primary-school children, evaluating a physically active learning intervention, comparing the intervention with usual teaching or another eligible control condition, and reporting at least one prespecified physical activity, sedentary-behaviour, physical-fitness, motor-competence, or body-composition outcome. There was no time limit for database searches in this study. Only English-language literature was included. Review articles, conference proceedings, commentaries, and other language literature, as well as other materials unrelated to this study, were not included. The population included children aged 6–12 years who were enrolled in primary or elementary school at baseline. Studies with a wider age range were eligible only when data for children aged 6–12 years were reported separately or when the primary-school subgroup could be identified. Studies were not excluded solely on the basis of sex, ethnicity, socioeconomic background, obesity, or health status unless the condition prevented participation in the intervention or valid assessment of the outcomes.

The intervention had to meet the operational definition of Physically Active Learning: purposeful physical activity integrated into the delivery or practice of academic curriculum content. Eligible interventions included physically active mathematics, literacy, science, or other academic lessons, as well as curriculum-linked movement activities. General physical-education classes, recess-only programmes, active commuting, extracurricular sport, sports training, nutrition-only interventions, and movement breaks without an academic-learning component were excluded. The intervention had to last at least 6 weeks. Frequency, session duration, academic subject, activity type, and intensity were extracted for each study; no single minimum weekly frequency was imposed because PAL programmes differ in their curriculum design.

Eligible activities included age-appropriate walking, stepping, running, jumping, dance, balance, coordination, agility, strength, muscular-endurance, classroom-circuit, exercise-game, aerobic, and other motor activities when they were integrated with academic instruction. The activity did not need to reach moderate-to-vigorous intensity for study eligibility, but intensity was extracted and considered when interpreting the findings.

The comparator had to be a concurrent control condition that did not receive the PAL component during the comparison period. Eligible controls included usual classroom teaching, the usual school curriculum, delayed-treatment groups, wait-list groups, and clearly defined active comparators. The presence of ordinary physical education or recess was recorded because these activities could influence the intervention contrast. Comparisons based only on geographic region, ethnicity, boarding status, school type, or academic module were not considered eligible intervention comparators unless they formed part of a controlled PAL study.

Eligible outcomes included physical activity, moderate-to-vigorous physical activity, sedentary behaviour, cardiorespiratory fitness, muscular fitness, motor competence, agility or speed, flexibility, body composition, BMI, body mass, or waist circumference. The measurement instrument, device, questionnaire, unit, observation window, and follow-up time were extracted for every outcome. Minutes, percentages, steps, activity counts, questionnaire scores, fitness-test scores, and anthropometric measures were retained in their original formats and were not treated as interchangeable.

Eligible study designs included randomized controlled trials, cluster-randomized controlled trials, crossover trials, controlled clinical trials, controlled before–after studies, and quasi-experimental studies with a concurrent comparator. Descriptive, cross-sectional, uncontrolled, qualitative-only, protocol, review, editorial, commentary, and conference studies without sufficient outcome data were excluded.

#### 2.1.2. Research Selection

**Figure 1:**
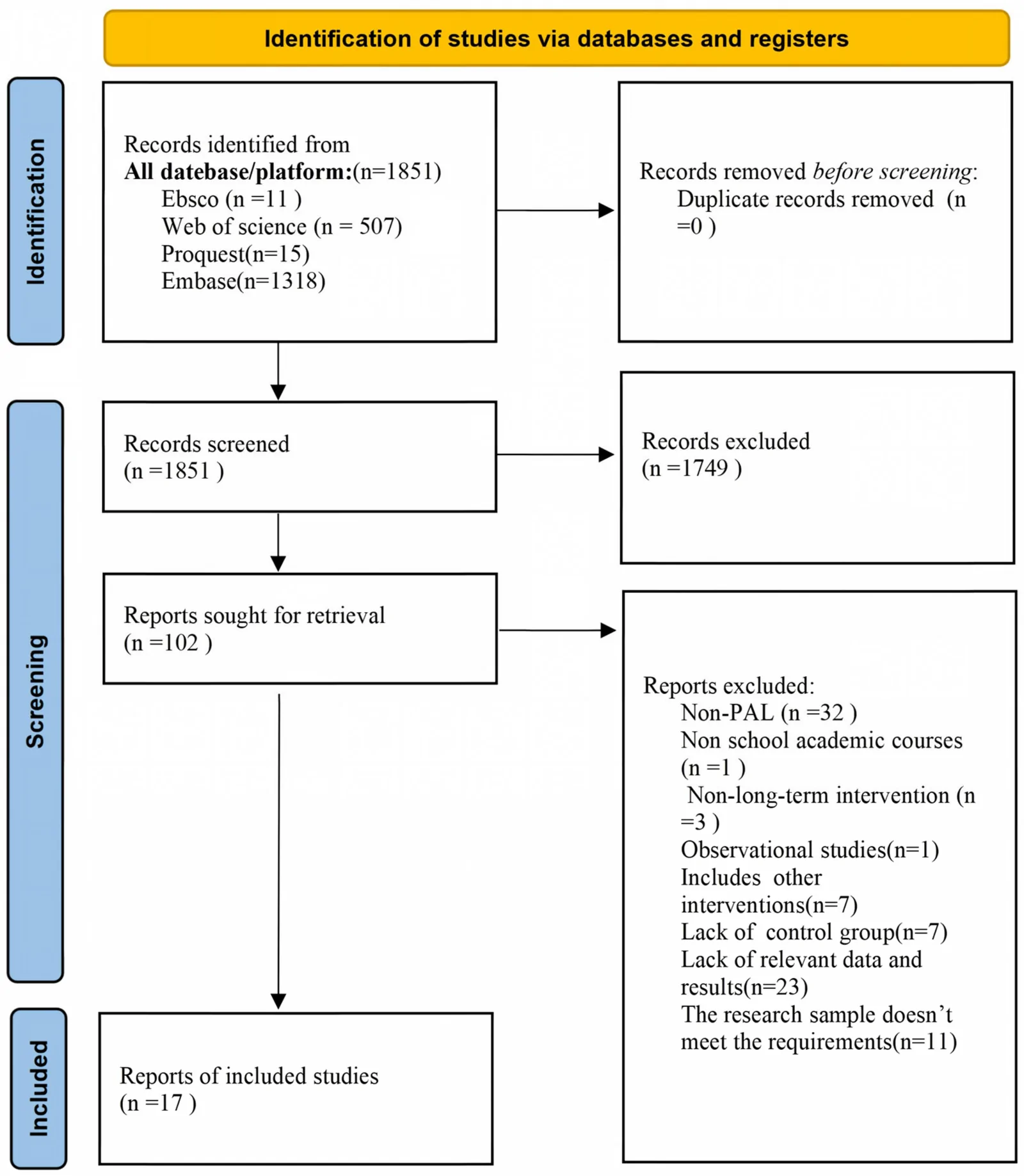
PRISMA flow diagram for the systematic review.

Our search strategy was based on the PRISMA guidelines (Supplementary Materials), and the detailed process is shown in Figure 1. The search formula included the following keywords: (“primary school” OR “elementary school” OR “primary education” OR “junior school”) AND (“physically active learning” OR “active classroom” OR “active academic lesson” OR “movement integration” OR “physically active academic lesson” OR “acute exercise” OR “acute physical activity”) AND (“children” OR “child” OR “young” OR “School-aged student” OR “adolescent” OR “teenager”) AND (“physical Fitness” OR “cardiorespiratory fitness” OR “aerobic fitness” OR “MVPA” OR “muscular fitness” OR “motor skills” OR “body composition” OR “health” OR “sedentary” OR “motor competence”). We searched four electronic databases and platforms: Web of Science, ProQuest, Ebsco, and Embase. On the ProQuest platform, we searched the Psychology Collection and Sports Medicine & Education index (Tables S1–S4). On the Ebsco platform, we searched CINAHL Ultimate, MEDLINE Complete and ERIC. Web of Science, ProQuest, and Ebsco use subject term searching, while Embase uses All fields searching. In the Embase database, we retrieved 1084 records of articles. Moreover, our search period started from the database inception and ended on 1 June 2026. In total, 1613 relevant studies were retrieved. Subsequently, review articles, conference proceedings, commentaries, non-English-language publications, and other materials unrelated to this review were excluded based on the title, keywords, and abstract. A total of 1511 records were excluded. Finally, 102 studies were included for detailed analysis. Each study was reviewed and screened based on its research subjects, methods, and results. According to the inclusion criteria and screening conditions, articles using samples that did not meet the predefined inclusion criteria were excluded. In the end, this study included 17 articles.

#### 2.1.3. Study Selection

Search results were exported from each database in RIS format and imported into EndNote. Duplicate records were identified using EndNote’s duplicate-detection function and were subsequently checked manually using title, first author, year, journal, and DOI. The deduplicated library was backed up before screening. Pre-defined inclusion and exclusion criteria were then applied to titles and abstracts, and studies potentially meeting the inclusion criteria were evaluated in full. If disagreements arose regarding eligibility for inclusion, two other researchers discussed the study in detail to reach a consensus based on the established criteria. Final inclusion decisions were made through collaborative discussion and agreement. All extracted data were independently cross-checked to ensure accuracy and consistency.

#### 2.1.4. Data Extraction

Two reviewers, [JB] and [TV], independently screened titles and abstracts against the predefined eligibility criteria. Potentially eligible records were retrieved in full text and independently assessed by both reviewers. Disagreements were resolved through discussion; unresolved disagreements were adjudicated by [MB]. The reasons for full-text exclusion were recorded using one primary reason per report. Extracted information included authors, publication year, country/region, participants’ age, sex, sample size, and outcome measures related to PAL, physical activity, physical fitness, sedentary behavior, and other relevant health indicators, as well as details of interventions (e.g., frequency and duration of interventions). We also documented the key indicators involved in these studies and the changes in these indicators.

#### 2.1.5. Risk of Bias

**Figure 2:**
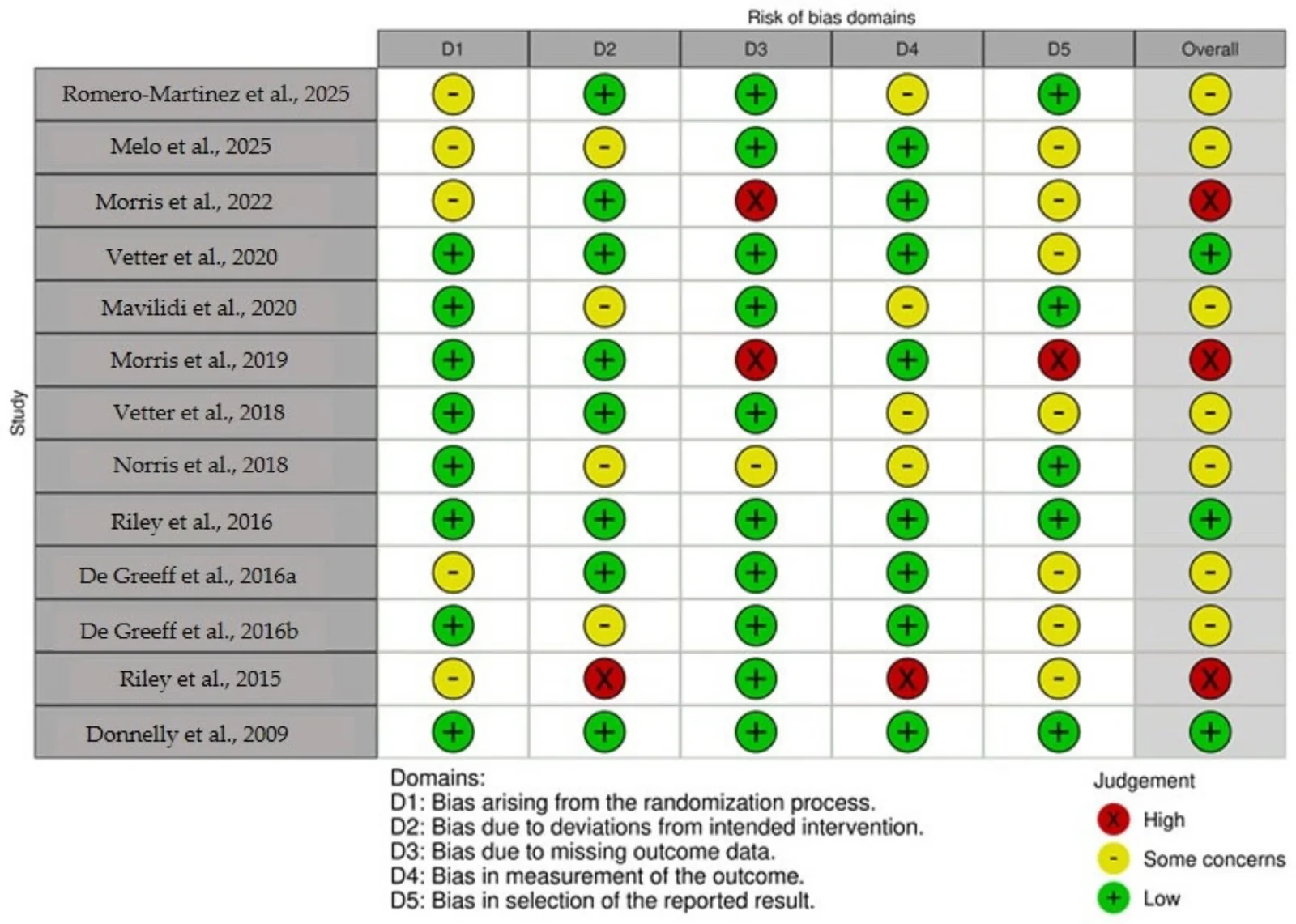
ROB2 bias by domain and overall bias for each study [11,12,13,14,15,16,17,18,19,20,21,22,23].

**Figure 3:**
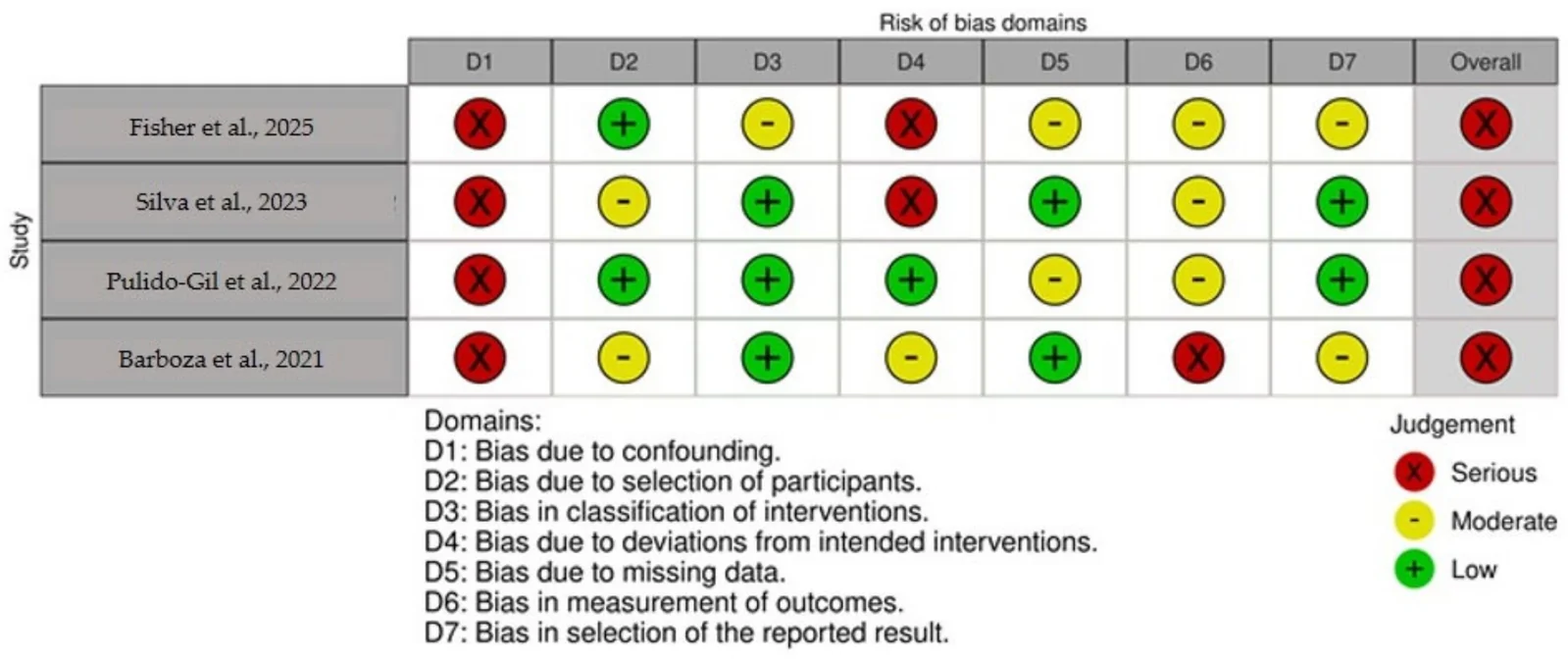
ROBINS-I bias by domain and overall bias for each study [24,25,26,27].

Two reviewers independently assessed the risk of bias for each included study, and resolved any differences through discussion; if no consensus could be reached, the final decision was made by a third reviewer. For randomized controlled trials, we used the ROB2 tool for assessment (Figure 2); for non-randomized controlled trials, we used the ROBINS-I tool for assessment (Figure 3). In total, 13 randomized controlled trials and 4 non-randomized controlled trials were included. The overall methodological quality of the randomized controlled trials was moderately low. Only 3 studies were rated as having low bias risk, while the remaining 10 studies all had a high or moderate level of bias risk. Among them, the randomization process (D1) was the better domain. Non-randomized studies were considered to have varying degrees of risk in 7 ROBINS-I domains (including confounding factors, subject selection, intervention classification, intervention deviation, missing data, outcome measurement, and selective reporting), and the overall assessment of 4 studies was serious risk. Overall, the evidence quality was essentially lower than that of randomized controlled trials. In conclusion, the core conclusion of this review is mainly based on moderate to low-quality evidence. Due to the lack of blinding of the intervention measures, the results of randomized controlled trials (RCTs) may overstate their effects, so they should be interpreted with caution. Non-randomized controlled studies are only used as supplementary evidence to assist in discussing the potential impact of the intervention measure in the real world.

## 3. Results

### 3.1. Descriptive Analysis—Key Features of PAL Intervention

This review analyzed 17 included studies. These studies explored the effects of school-based PAL interventions on children’s physical fitness, physical activity, and sedentary behavior. All the studies included were interventional trials with control groups, according to our PICOS design. We extracted and summarized the following key elements: authors and publication year; sample age; sample sex; sample size; intervention period; intervention frequency; intervention content in the experimental group; intervention content in the control group; and some key research indicators related to children’s physical fitness, physical activity and sedentary behavior.

Table 2 lists some key characteristics of the included studies.

The included studies were published between 2009 and 2025, and studies came from 8 different countries. The most common study design was the randomized controlled trial. All study samples include both boys and girls, and students’ ages are concentrated between 6 and 12 years old. The study with the largest sample size had 1527 participants [23], and the study with the smallest sample size had 50 students, with 25 in the intervention group and 25 in the control group [26]. There were 8 studies with an intervention period of 6 weeks [13,14,15,16,17,18,19,22]. Regarding the frequency of intervention, three studies intervened five times a week, meaning daily interventions were conducted on weekdays [11,12]; nine studies intervened three times a week [14,15,17,18,19,20,21,22,27]. Three other studies did not specify the exact number of interventions per week [16,23,25].

This review focuses on the impact of interventions that integrate daily school curriculum with physical activity on children’s physical fitness, physical activity, and sedentary behavior. Non-curricular activities were excluded. Intervention duration and frequency varied according to study design and implementation procedures. Because the included studies differed substantially in outcome definitions, measurement instruments, settings, follow-up periods, intervention doses, and statistical reporting, a structured narrative synthesis without meta-analysis was conducted. Studies were grouped by physical-activity, sedentary-behaviour, and physical-fitness domains and, where relevant, by lesson-level, school-day, or broader daily measurement context. Findings were summarized using the reported direction of effect, numerical magnitude when available, statistical significance, study design, and measurement limitations. Results reported in different units or from different observation windows were not averaged or combined. Table 3 summarizes the changes in key measures and outcomes across the selected reviewed studies.

### 3.2. Effects of PAL on Target Outcomes

#### 3.2.1. Physical Activity Levels

Physical activity was reported as moderate-to-vigorous physical activity (MVPA), light physical activity (LPA), or step counts; however, the studies differed in measurement instruments, observation periods, intervention settings, and outcome units. MVPA was interpreted according to the measurement protocol and intensity thresholds used in each original study. Because the studies did not use a common measurement context, results obtained during PAL or mathematics lessons were considered separately from school-day, daily, or weekly outcomes. A total of 12 studies mentioned physical activity. Among them, 6 studies indicated significant differences in the duration of moderate-to-vigorous physical activity (MVPA) [11,13,14,18,19,22]. However, it is worth noting that due to different experimental designs, the results of some studies varied slightly. For example, Norris et al.’s study only indicated significant differences in MVPA during class time, while no significant changes were observed at other times [18]. Riley et al.’s study also revealed differences in MVPA during math class [19,22]. This suggests to some extent that experimental interventions can increase students’ MVPA time during class. However, four other studies reported no significant between-group differences in moderate or vigorous physical activity [15,16,25,27], indicating that the effect was not consistent across intervention designs or measurement periods. Regarding the results of light physical activity (LPA), four studies mentioned statistically significant temporal interactions in LPA among the participants [16,22,25,27]. Three studies mentioned no significant differences in LPA [13,18,19]. It can be seen that MVPA and LPA show different results in different intervention experiments, which is related to the fact that there is no unified standard for the current PAL curriculum design. In addition, two other studies also measured students’ daily walking data during school. One study showed no significant difference in daily and weekly walking data [12], while the other study showed a significant increase in students’ step count [14].

#### 3.2.2. Sedentary Behavior

Seven studies assessed sedentary behaviour or closely related classroom activity patterns. Sedentary behaviour was evaluated using sedentary minutes, sitting time, stationary time, or the percentage of time spent sedentary. Because the studies used different observation periods, outcomes were reported separately for the PAL lesson, mathematics lessons, breaks, the school day, weekdays, and broader daily periods rather than being combined into a single average. In evaluating the direct effect of PAL on sedentary time, our analysis reveals a distinct pattern, addressing a specific gap in empirical evidence where direct effects remain sparsely documented. Overall, most studies reported a favourable effect of PAL on at least one sedentary-behaviour or activity-pattern outcome. Norris et al. analyzed the results of sedentary behavior on school days, weekdays and between classes, and found no significant difference in sedentary behavior on school days and weekdays, but a significant reduction in sedentary behavior during breaks [18]. Riley et al. reported that the percentage of students sitting during math class decreased significantly, while no significant difference was observed in the results throughout the day [19]. In another study, Riley et al. also reported on sedentary data during school days and math class, and the results showed a significant decrease in the results for both groups [22]. Morris et al. reported on sedentary time, and the results showed no significant difference between groups [16]. Regarding other health-related indicators, Silva et al. measured variables such as standing time, walking time, number of sit–stand transitions and rest time, and the results showed that the time interaction between groups was statistically significant, while there was no significant difference in sitting time between groups [25]. Barboza et al. also showed similar results, showing no significant difference in sitting time [27].

#### 3.2.3. Physical Fitness

Eight studies assessed at least one physical-fitness or body-composition outcome. Physical fitness was treated as a multidimensional construct comprising motor competence, cardiorespiratory fitness, muscular fitness, agility or speed, flexibility, and body composition. Motor competence was assessed using Actual Motor Competence measures, whereas cardiorespiratory fitness was assessed using VO_2_max or field-based endurance tests measuring distance or levels. Muscular fitness included curl-up, sit-up, push-up, handgrip, and related strength or endurance tests. BMI was treated as a body-composition indicator rather than as a direct measure of physical fitness. Because these tests assess different physiological and motor domains, their results were reported separately and were not combined into a single overall fitness score. The results of Romero et al. showed that the improvement in Actual Motor Competence (Actual MC) in the experimental group after the test was significantly greater than the improvement in the pretest [11]. No significant interaction between Perceived MC (motor competence) and Perceived PL (physical literacy) was reported in the article. This indicates to some extent that there is a difference between the individual’s perception of motor behavior and the actual measurement results. Four studies assessed BMI, and none reported a statistically significant between-group difference [14,21,23,24]. However, according to the results of Pulido Gil et al., the weight of students in the experimental group increased significantly, but there was no significant difference in waist circumference [26]. This should be interpreted as a normal growth and development phenomenon in children. Regarding aerobic capacity, the results of Fisher and Africa showed that there was no significant improvement compared to the control group [24]. Two other studies showed that the intervention improved students’ VO_2_peak [14,17]. In addition, there are articles showing that PAL intervention can play a certain role in improving students’ muscle strength and other aspects. For example, one study showed that the experimental group had significantly improved curl-up and push-up performance [24], but two studies showed no significant difference in students’ sit-up performance [20,21]. In addition, the experiment also improved students’ long jump and endurance, with significant improvements [26]. There were no significant changes in indicators of athletic ability such as 10 × 5 m agility and 20 m shuttle run [20,21].

## 4. Discussion

This systematic review included 17 studies examining the effects of school-based PAL interventions on physical activity, physical fitness, and sedentary behavior among primary school students. Some studies reported significant increases in MVPA [11,13,14,18,19,22], while others found no significant changes in MVPA [15,16,25,27]. This difference may be closely related to variations in intervention design, including intervention frequency, duration, content, and measurement tools, leading to different outcomes. For example, Norris et al. observed significant improvements in MVPA only in the classroom, with no changes in MVPA throughout the day or on weekends [18]. This may suggest that the effects of PAL may be primarily limited to classroom time and insufficient to alter overall daily activity levels. Moreover, the findings indicate that activity outcomes are shaped by multiple contextual domains—including the school, family and peers, community environment, and policy—rather than by a single setting alone [28].

The effects of PAL interventions on BMI were not significant [14,21,23,24]. The included studies generally evaluated PAL without additional intervention components, and changes in BMI require longer intervention periods or combinations with dietary control, which may explain the relatively small differences in BMI values in this study. This possibility is important because dietary behavior and sedentary behavior develop alongside physical-activity habits during childhood and adolescence, and the health consequences of sedentary lifestyles cannot be addressed through activity promotion alone [29]. PAL interventions mainly consist of physical activities aligned with curriculum requirements, lacking specific targeted training in motor skills. Improvements in abilities such as flexibility and aerobic capacity may require more relevant exercise stimulation; therefore, different experiments yielded different results regarding the effects on these indicators. Furthermore, most of the interventions included in this study were short-term and unlikely to have a significant impact on weight management and exercise indicators.

PAL interventions have shown relatively consistent effects in reducing sedentary behavior in the classroom. Multiple studies have reported that standing time and walking time have increased, while sitting time or prolonged sitting time has decreased significantly. This suggests that PAL can effectively reduce children’s sedentary behavior, thereby improving classroom behavior patterns. In addition, Norris et al. found that although there was no significant change in sedentary behavior throughout the day, sedentary behavior in the classroom was significantly reduced, which also shows that the effect of PAL intervention varies depending on the measurement environment [18].

Our findings confirm that PAL interventions can improve physical activity levels in the classroom. However, their impact on fitness indicators (BMI, cardiorespiratory health) is limited. Admittedly, we cannot simply assert that PAL interventions have a significant positive impact on children’s physical health, but their effectiveness in reducing sedentary behavior is perhaps the most noteworthy aspect. This suggests that PAL should be considered a complementary component of a school’s strategy to promote physical activity, rather than the sole means. Schools and education administrators should explore the synergistic effects of PAL with other opportunities for physical activity throughout the day, including active breaks, physical education classes, active travel to and from school, and extracurricular activities involving greater levels of physical activity. In addition, the family environment should be included in this strategy. In most recent research, parent–child sedentary behavior was associated among insufficiently active mothers and fathers, whereas the association was not statistically significant among physically active parents [30]. Combining these approaches may cumulatively increase students’ overall daily physical activity, thereby maximizing the benefits of exercise, improving physical fitness, and promoting both physical and mental health.

### Practical Implications

Rather than viewing PAL as a standalone solution for children’s physical inactivity, schools and physical educators should conceptualize PAL as one core component of a multi-component school-based physical activity framework (e.g., the Comprehensive School Physical Activity Program, or CSPAP). To maximize health benefits, schools should seek synergistic relationships between PAL, active recess, active travel to and from school, extracurricular activities involving physical activity, and structured physical education classes when organizing the school day. Particular attention should also be given to the quality, composition, and organization of children’s meals provided at school. Combining these approaches is essential for helping students accumulate the WHO-recommended 60 min of daily moderate-to-vigorous physical activity, thereby promoting meaningful improvements in cardiovascular fitness, motor competence, body composition, and overall physical and mental health.

## 5. Limitations and Future Directions

Several key limitations within the current body of literature and the present review must be acknowledged. Beyond the constraints identified in the primary sources—such as a lack of diverse health-related biomarkers (e.g., blood pressure, blood lipids, metabolic profiles, and psychological well-being), short intervention windows (typically 6 to 8 weeks) lacking long-term follow-up assessments, and pronounced protocol heterogeneity in PAL curriculum design, lesson intensity, and outcome measurement tools—the present synthesis itself is bounded by inherent methodological constraints. Most notably, a primary limitation of this work is the relatively small number of studies that met our inclusion criteria; while stringent selection criteria were necessary to preserve analytical rigor, this modest pool of evaluated literature limits statistical power, restricts granular subgroup analyses, and warrants caution when generalizing these findings to broader pediatric populations. Furthermore, reliance on varying measurement instruments, such as non-standardized accelerometer wear times and cut-points across the available studies, introduces unaccounted heterogeneity, while restricting our search to published English-language literature leaves open the potential for publication bias. Additionally, a methodological limitation of this review is that the protocol was not prospectively registered in an international database such as PROSPERO. Future research should therefore prioritize large-scale, prospective cohort studies with standardized, device-based tracking and broader biomarker profiles to further validate and extend these empirical insights.

Moreover, future research on physically active learning (PAL) in schools should prioritize the development of validated, standardized, and openly accessible intervention protocols with clearly defined recommendations regarding activity type, intensity, duration, frequency, and implementation conditions. Large-scale, multicenter cluster-randomized controlled trials are needed to improve the methodological quality and generalizability of findings, while follow-up assessments conducted 6–12 months after the intervention should be included to determine whether positive behavioral and health-related effects are maintained over time. Future studies should also broaden their outcome measures by combining objective assessments of physical activity and physical fitness with cognitive testing, standardized academic achievement measures, detailed monitoring of school performance, mental health indicators, motor competence, body composition, and metabolic health markers. In addition, researchers should consider the influence of active recess, active travel to and from school, extracurricular physical activity, physical education, sedentary time, sleep, and the quality and organization of children’s nutrition at school. Such comprehensive and interdisciplinary research designs would provide a more robust understanding of the effectiveness, feasibility, sustainability, and wider educational and health-related impact of PAL in school settings.

## 6. Conclusions

This systematic review demonstrates that PAL interventions effectively enhance moderate-to-vigorous physical activity and reduce sedentary behavior within primary school classrooms during target academic lessons. However, these positive behavioral adjustments remain primarily lesson-specific and do not consistently extend to whole-school-day physical activity levels or daily sedentary time. Furthermore, while PAL yields targeted improvements in specific physical fitness components—such as motor competence, upper-body muscular endurance, and cardiorespiratory health—its overall impact on body mass index remains equivocal. Consequently, PAL should not be viewed as a standalone solution for pediatric physical inactivity or fitness development. To maximize its health and educational benefits, PAL should be strategically integrated within a comprehensive, whole-school physical activity framework alongside structured physical education, active recess, active commuting, and supportive family environment initiatives.

## Figures and Tables

**Table 1:** PICOS framework.

PICOS Element	Main Eligibility Criterion
Population (P)	Primary-school children aged 6–12 years at baseline.
Intervention (I)	Physically Active Learning intervention integrating purposeful physical activity with academic curriculum content for at least 6 weeks.
Comparator (C)	Concurrent usual teaching, usual school curriculum, delayed-treatment, wait-list, or clearly defined active comparator without the PAL component.
Outcomes (O)	At least one physical-activity, sedentary-behaviour, physical-fitness, motor-competence, or body-composition outcome measured quantitatively.
Study design (S)	Randomized, cluster-randomized, crossover, controlled clinical, controlled before–after, or quasi-experimental studies with a concurrent comparator.

**Table 2:** Characteristics of intervention samples.

Author/Years	Country	Study Design	Age	Gender	Sample Size	Intervention Duration	Intervention Frequency	Intervention Time
Romero-Martínez et al., 2025 [11]	Spain	RCT	10–11	B & G	E: 3 school CON: 3 school All students: 332	9–10 weeks	5 times/week	No PE classes: 30 min (3 items) There are PE classes: 2 items
Melo et al., 2025 [12]	Brazil	Cluster randomized trials	6.9 ± 0.6	B & G	PAL group: 77 AB group: 61 CTL groups: 46	8 weeks	5 times/week	Once 30–50 min
Fisher & Africa 2025 [24]	South Africa	Quasi experimental	6–8	B & G	E: 68 CON: 51	16 weeks	2 times/week	Once 30 min
Silva et al., 2023 [25]	Brazil	Controlled clinical trial with cluster sampling	7.8 ± 0.6	B & G	E: 34 CON: 27	2 years	/	/
Pulido Gil et al., 2022 [26]	Spain	Quasi experimental	10.62 ± 0.57	B & G	E: 25 CON: 25	8 weeks	1 time/week	**/**
Morris et al., 2022 [13]	United Kingdom	RCT	9.61 ± 0.29	B & G	E: 51 CON: 46	6 weeks	1 time/week	50 min
Barboza et al., 2021 [27]	Brazil	Cluster controlled trial	7.8 ± 0.6	B & G	E: 34 CON: 27	9 months	3 times/week	Once 15 min
Vetter et al., 2020 [14]	Australia	Randomized controlled cross-over trial	8.4 ± 0.3	B & G	Classroom: 170 Playground: 70 All samples: 170	6 weeks	3 times/week	Once 30 min
Mavilidi et al., 2020 [15]	Australia	RCT	9.81 ± 0.68	B & G	TWM-E: 121 Control: 162	6 weeks	3 times/week	Once/40 min
Morris et al., 2019 [16]	United Kingdom	Cluster randomized controlled trial	9.9 ± 0.3	B & G	E: 52 CON: 31	6 weeks	/	/
Vetter et al., 2018 [17]	Australia	Randomized crossover trial	9.8 ± 0.3	B & G	PA: 85 Classroom: 85 All samples: 85	6 weeks	3 times/week	Once 20 min
Norris et al., 2018 [18]	United Kingdom	Cluster randomized controlled trial	8–9	B & G	VT group: 113 COM group: 106	6 weeks	3 times/week	Once 10 min
Riley et al., 2016 [19]	Australia	Clustered randomized controlled trials	11.1 ± 0.7	B & G	E: 142 CON: 98	6 weeks	3 times/week	Once 60 min
de Greeff et al., 2016 [20]	Netherlands	Randomized controlled trial	8.1 ± 0.7	B & G	E: 249 CON: 250	2 years	3 times/week	Once/20–30 min
de Greeff et al., 2016 [21]	Netherlands	cluster-randomized controlled trial	8.1 ± 0.7	B & G	E: 181 CON: 195	2 years	3 times/week	Once 30 min
Riley et al., 2015 [22]	Australia	Randomised controlled trial	10.53 ± 0.7	B & G	E: 27 CON: 27	6 weeks	3 times/week	Once 60 min
Donnelly et al., 2009 [23]	US	cluster randomized controlled trial	PAAC: 7.7 ± 0.4 CON: 7.8 ± 0.4	B & G	PAAC: 814 CON: 713	3 years	/	90 min/week

**Note**: **E**—experimental; **CON**—control; **B**—boys; **G**—girls; **n**—number; **min**—minutes; **RCT**—Randomized Controlled Trial; **PAL**—Physically active learning; **MVPA**—moderate to vigorous physical activity; **PL**—physical literacy; **MC**—motor competence; **BMI**—body mass index; **PE**—physical education; **PA**—physical activity; **STS**—sit-to-stand; **CARS**—Children’s Activity Rating Scale; **MPA**—moderate physical activity; **VPA**—vigorous physical activity; **LPA**—light physical activity; **SB**—sedentary behavior; **CPM**—counts per minute; **SBJ**—standing broad jump; **SUP**—sit-ups; **HG**—handgrip strength; **10 × 5 m SR**—10 × 5 m shuttle run; **20 m SR**—20 m endurance shuttle run; **↑**—increased (↑*—significant increased); **↓**—decreased (↓*—significant decreased) **↔**—no significant difference.

**Table 3:** Intervention content and results.

Author/Years	E Group	CON	Main Indicator	Results
Romero-Martínez et al., 2025 [11]	PAL	Regular teaching and learning	MVPA; Actual MC	↑* MVPA (PAL 46.8–54.1; CON 32.7–38.3); ↑* Actual MC
Melo et al., 2025 [12]	PAL	usual lessons	At school (step/day); In the week (step/day); Physical activity (frequency)	**↔ -**At school (step/day); **↔** In the week (step/day); **↔** Physical activity (frequency)
Fisher & Africa 2025 [24]	Physically Active Academic Intervention	No intervention	BMI; Aerobic capacity; Curl-up; Push-up; Trunk-lift; Sit-and reach	**↔** BMI; **↔** Aerobic capacity; ↑* Curl-up (PAL 1.43–6.16; CON 1.81–1.88); ↑* Push-up (PAL 0.85–6.22; CON 1.29–1.17); **↔** Trunk-lift; **↔** Sit-and reach
Silva et al., 2023 [25]	Physically active lessons	No intervention	Sitting time; Stationary time; Walking time; LIPA time; Standing time; STS transitions; MPA time; VPA time	**↔** Sitting time; ↓* Stationary time; ↑*-Walking time; ↑* LIPA time; ↓* Standing time; ↑*-STS transitions; **↔** MPA time; **↔** VPA time
Pulido Gil et al., 2022 [26]	PAL	No intervention	Step count; Handgrip strength; Broad jump; 20 m SR	↑* Step count (3331–3720) **↔**Handgrip strength (17.4–17.7 kg); ↑* Broad jump (134–146 cm); ↑ 20 m SR (230.6–277.2 m)
Morris et al., 2022 [13]	PAL	No intervention	Sedentary Minutes; LPA Minutes; MVPA Minutes	**↔** SB (PAL −27; CON −18); **↔** LPA (PAL +19.8; CON +14.1); ↑* MVPA (PAL +6.4; CON +1.4)
Barboza et al., 2021 [27]	Physically active lessons	No additional physical activity	Sitting time; Standing time; Walking time; Stationary time; Light PA time; Moderate to vigorous PA time	**↔** Sitting (PAL −0.4; CON 1.3); ↓* Standing (PAL −3.4; CON −1.8); ↑* Walking (PAL 3.9; CON 0.4); ↓* Stationary (PAL 12.6; CON 7.0); ↑* LPA (PAL 9.4; CON 5.0); **↔** MVPA (PAL 5.0; CON 4.3)
Vetter et al., 2020 [14]	Physically active lessons in playground	Classroom Lesson Games	VO_2_peak; BMI; MVPA; Vigorous; Step Counts	↑* VO_2_peak; **↔** BMI; ↑* MVPA (PAL 12.3; CON 0.9); ↑* Vigorous; ↑* Step Counts
Mavilidi et al., 2020 [15]	Physically active lessons	Normal curricular lessons	MVPA; Moderate PA; Vigorous PA	**↔** MVPA (PAL 51.1–52.8; CON 44.9–46.9); **↔** Moderate PA; **↔** Vigorous PA
Morris et al., 2019 [16]	PAL	No intervention	MVPA; LPA; Sedentary Time	**↔** MVPA (PAL 6.1–6.4%; CON 6.7–6.8); ↑* LPA (PAL 29.8–31.8; CON 29.9–29.8); **↔** SB (PAL 64.1–61.8; CON 63.8–63.2)
Vetter et al., 2018 [17]	Physical activity intervention	Classroom lesson games	VO_2_peak	↑* VO_2_peak
Norris et al., 2018 [18]	Physically active lesson intervention	typical teaching	School day MVPA; Weekend day MVPA; School day SB; School day LPA; Weekend day SB; Weekend day LPA; Lesson SB; Lesson LPA; Lesson MVPA; CARS Lesson PA	↑* School day T1 MVPA (PAL 60.8; CON 56.1); **↔** Weekend day MVPA; **↔** School day SB; **↔** School day LPA; **↔** Weekend day SB; **↔** Weekend day LPA; ↓* Lesson SB; **↔** Lesson LPA; ↑* Lesson MVPA; **↔** CARS Lesson PA
Riley et al., 2016 [19]	PAL	regular lessons	MVPA day%; MVPA Math%; SB day%; SB Math%; LPA day%; LPA Math%; Av min MVPA day; Av min MVPA Math;	**↔** MVPA day% (1.7 diff; *p* = 0.52); ↑* MVPA Math% (2.6 diff); ↓* SB day% (−3.5 diff) ↓* Math SB% (−8.2 diff); ↔ LPA day% (1.8 diff); ↑* LPAMath% (5.5 diff); **↔** Av min MVPA day (PAL 30.9–33.3; CON 27.8–29.5); ↑* Av min MVPA Math (PAL 2.2–3.6; CON 1.8–1.6);
de Greeff et al., 2016 [20]	Physical activity across the curriculum	No intervention	10 × 5 m SR; 20 m SR; SBJ; SUP; HG	**↔** 10 × 5 m SR; **↔** 20 m SR; **↔** SBJ; **↔** SUP; ↑* HG
de Greeff et al., 2016 [21]	Physical activity across the curriculum	No intervention	BMI; 10 × 5 m SR; 20 m SR; SBJ; SUP; HG	**↔** BMI; **↔** 10 × 5 m SR; **↔** 20 m SR; **↔** SBJ; **↔** SUP; **↔** HG
Riley et al., 2015 [22]	Integrating PA lessons	No intervention	School day SB; School day LPA; School day MVPA; Math class SB; Math class LPA; Mathematics class MVPA;	↓* School day SB (PAL −13.9; CON 4.7); ↑* School day LPA (PAL 10.9; CON −2.0); ↑* School day MVPA (PAL 3.0; CON −5.7); ↓* Math class SB (PAL −19.9; CON 2.6); ↑* Math class LPA (PAL 9.1; CON −3.6); ↑* Math class MVPA (PAL 10.7; CON 1.0)
Donnelly et al., 2009 [23]	Physically active academic lessons	Regular classroom lessons	BMI	**↔ --**BMI

**Note**: **E**—experimental; **CON**—control; **B**—boys; **G**—girls; **n**—number; **min**—minutes; **RCT**—Randomized Controlled Trial; **PAL**—Physically active learning; **MVPA**—moderate to vigorous physical activity; **PL**—physical literacy; **MC**—motor competence; **BMI**—body mass index; **PE**—physical education; **PA**—physical activity; **STS**—sit-to-stand; **CARS**—Children’s Activity Rating Scale; **MPA**—moderate physical activity; **VPA**—vigorous physical activity; **LPA**—light physical activity; **SB**—sedentary behavior; **CPM**—counts per minute; **SBJ**—standing broad jump; **SUP**—sit-ups; **HG**—handgrip strength; **10 × 5 m SR**—10 × 5 m shuttle run; **20 m SR**—20 m endurance shuttle run; **↑**—increased (↑*—significant increased); **↓**—decreased (↓*—significant decreased) **↔**—no significant difference.

## Data Availability

The data are available upon reasonable request from the authors.

## Supplementary Materials

The following supporting information can be downloaded at https://www.mdpi.com/article/10.3390/children13091267/s1, Table S1: Search strategy for Embase (1084); Table S2: Search strategy for ProQuest (6) Database: Psychology Collection; Sports Medicine & Education index; Table S3: Search strategy for Web of Science Core Collection (513); Table S4: Search strategy for EBSCOhost (10) Database: CINAHL Ultimate; MEDLINE Complete; ERIC; PRISMA 2020 Checklist.
