# Supplementary material for: Effects of Physically Active Learning (PAL) Interventions on Physical Fitness, Physical Activity, and Sedentary Behavior in Primary School Children: A Systematic Review
Source: Children (Basel). 2026 Sep 18;13(9):1267. doi: 10.3390/children13091267 (PMC13605041; doi:10.3390/children13091267)
Supplement: Supplementary file 1 [file children-13-01267-s001.zip › children-4490282-supplementary.pdf]

Supplementary Materials

Appendix 1 Search strategy

Table S1. Search strategy for Embase (1084).

Database/platform	Date(year-month-day)
Embase	Start to End:<br>1883-01-01 to 2026-06-01

(primary AND school OR (elementary AND school) OR (primary AND education) OR (junior AND school)) AND (physically AND active AND learning OR (active AND classroom) OR (active AND academic AND lesson) OR (movement AND integration) OR (physically AND active AND academic AND lesson) OR (acute AND exercise) OR (acute AND physical AND activity)) AND (children OR child OR young OR ('school aged' AND student) OR adolescent OR teenager) AND (physical AND fitness OR (cardiorespiratory AND fitness) OR (aerobic AND fitness) OR mvpa OR (muscular AND fitness) OR (motor AND skills) OR (body AND composition) OR health OR sedentary OR (motor AND competence)) AND [01-01-1883]/sd NOT [02-06-2026]/sd

Table S2. Search strategy for ProQuest（6）Database：Psychology Collection ；Sports Medicine & Education index.

Database/platform	Date(year-month-day)
ProQuest	Start to End:<br>1900-01-01 to 2026-06-01

((subject(primary school) OR subject(elementary school) OR subject(primary education) OR subject(junior school)) AND (subject(physically active learning) OR subject(active classroom) OR subject(active academic lesson) OR subject(movement integration) OR subject(physically active academic lesson) OR subject(acute exercise) OR subject(acute physical activity)) AND (subject(children) OR subject(child) OR subject(young) OR subject(School-aged student) OR subject(adolescent) OR subject(teenager)) AND (subject(physical fitness) OR subject(cardiorespiratory fitness) OR subject(aerobic fitness) OR subject(MVPA) OR subject(muscular fitness) OR subject(motor skills) OR subject(body composition) OR subject(health) OR subject(sedentary) OR subject(motor competence)))

Table S3. Search strategy for Web of Science Core Collection (513).

Database/platform	Date(year-month-day)
Web of Science	Start to End:<br>1985-01-01 to 2026-06-01

[Topic](“primary school” OR “elementary school” OR “primary education” OR “junior school”) AND [Topic](“physically active learning” OR “active classroom” OR “active academic lesson” OR “movement integration” OR “physically active academic lesson” OR “acute exercise” OR “acute physical activity”) AND [Topic](“children” OR “child” OR “young” OR “School-aged student” OR “adolescent” OR “teenager”) AND [Topic](“physical fitness” OR “cardiorespiratory fitness” OR “aerobic fitness” OR “MVPA” OR “muscular fitness” OR “motor skills” OR “body composition” OR “health” OR “sedentary” OR “motor competence”)

Table S4. Search strategy for EBSCOhost (10)Database: CINAHL Ultimate; MEDLINE Complete；ERIC

Database/platform	Date(year-month)
EBSCOhost	Start to End:<br>1900-01 to 2026-06

[Subject Terms](“primary school” OR “elementary school” OR “primary education” OR “junior school”) AND [Subject Terms] (“physically active learning” OR “active classroom” OR “active academic lesson” OR “movement integration” OR “physically active academic lesson” OR “acute exercise” OR “acute physical activity”) AND [Subject Terms](“children” OR “child” OR “young” OR “School-aged student” OR “adolescent” OR “teenager”) AND [Subject Terms](“physical fitness” OR “cardiorespiratory fitness” OR “aerobic fitness” OR “MVPA” OR “muscular fitness” OR “motor skills” OR “body composition” OR “health” OR “sedentary” OR “motor competence”)
